# Response of a new rumen-derived *Bacillus licheniformis* to different carbon sources

**DOI:** 10.3389/fmicb.2023.1238767

**Published:** 2023-11-01

**Authors:** Yuchen Cheng, Jie Zhang, Wenyi Ren, Lili Zhang, Xiaofeng Xu

**Affiliations:** College of Animal Science and Technology, Ningxia University, Yinchuan, China

**Keywords:** *Bacillus licheniformis*, cows rumen fluid, different carbon sources, transcriptome, proteome

## Abstract

**Introduction:**

*Bacillus licheniformis* (*B. licheniformis*) is a microorganism with a wide range of probiotic properties and applications. Isolation and identification of novel strains is a major aspect of microbial research. Besides, different carbon sources have varying effects on *B. licheniformis* in regulating the microenvironment, and these mechanisms need to be investigated further.

**Methods:**

In this study, we isolated and identified a new strain of *B. licheniformis* from bovine rumen fluid and named it *B. licheniformis NXU98*. The strain was treated with two distinct carbon sources—microcrystalline cellulose (MC) and cellobiose (CB). A combination of transcriptome and proteome analyses was used to investigate different carbon source effects.

**Results:**

The results showed that *B. licheniformis NXU98* ABC transporter proteins, antibiotic synthesis, flagellar assembly, cellulase-related pathways, and proteins were significantly upregulated in the MC treatment compared to the CB treatment, and lactate metabolism was inhibited. In addition, we used MC as a distinct carbon source to enhance the antibacterial ability of *B. licheniformis NXU98*, to improve its disease resistance, and to regulate the rumen microenvironment.

**Discussion:**

Our research provides a potential new probiotic for feed research and a theoretical basis for investigating the mechanisms by which bacteria respond to different carbon sources.

## 1. Introduction

Since the discovery of penicillin in 1928, the use of antibiotics in humans and livestock production has become increasingly common (Butaye et al., 2003). Their use for improving livestock production, feed-energy conversion, and preventing the spread of infectious diseases has been effective (Dahiya et al., 2006). However, with the reckless use of antibiotics, antimicrobial resistance has steadily increased. With a decline in meat and egg production and increased infection rates in livestock and poultry, the public has begun to question the widespread use of antibiotics (Miciński et al., 2015). The EU’s new veterinary regulation (EU 2019/6) has extended the restrictions on the use of some antibiotics in animals to a full ban (Ivo, 2023). Therefore, the search for alternatives to antibiotics has become the focus of current research. In recent years, with the development of technology, bacteriocins (Cui et al., 2021), plant extracts (Bordean et al., 2023), antimicrobial vaccines (Rosini et al., 2020), and probiotics (Meng et al., 2022) have gradually emerged as new antibacterial drugs.

*Bacillus licheniformis* (*B. licheniformis*) is a Gram-positive cellulose-degrading bacterium belonging to the genus *Bacillus* (Muras et al., 2021; Yeak et al., 2022), capable of direct attachment to cellulose and crystalline cellulose, and is commonly found in natural environments such as soil or plant (Mondal et al., 2023; Nezafat et al., 2023). It exhibits functions such as the regulation of intestinal microbiota, growth promotion, anti-inflammatory and immunostimulatory effects, promotion of lipid profile modulation, increase in neurotransmitters, and stress reduction. It has physicochemical properties such as good stability, as well as resistance to oxidation, high temperature, acidic, and alkaline conditions, and secretes secondary metabolites with broad-spectrum antimicrobial activity, producing antimicrobial peptides that inhibit the growth of fungal or pathogenic microorganisms (Muras et al., 2021). As an efficient control bacterium, *B. licheniformis* is capable of reducing 94.7% of aflatoxin B1 and inducing the loss of aflatoxin B1 mutagenicity (Raksha et al., 2017; Wang et al., 2018).

However, the research on animal-origin *Bacillus* is insufficient. Screening strains from animals would enhance the bio-safety of food and animal feed. According to recent research, *B. licheniformis A-2-11B-AP* isolated from canine oral biofilms could significantly reduce the generation of dental caries by inhibiting the biofilm synthesis of the causative bacteria (Šurín Hudáková et al., 2022). Lei et al. (2023) isolated probiotics from yak feces and found that the isolated *Bacillus pumilus DX24* had a good survival rate and antibacterial activity that could increase daily gain and promote intestinal development in mice. Similarly, poultry probiotic bacillus demonstrated a positive effect on inhibiting *Escherichia coli* growth (Fernández et al., 2019).

As a probiotic, *B. licheniformis* is influenced by surrounding nutrients. Recent studies have shown that the same strain can survive under different carbon sources, each of which has different effects on the growth and metabolism of the strain and the accumulation of its metabolites (Castillo Alfonso et al., 2021; Ye et al., 2022), including deterring or inducing microbial enzyme production (Ba et al., 2023). Chang found that the metabolic capacity of different *Bacillus* species and related *Staphylococcus aureus* utilize multiple carbon sources for general metabolism that correlate with their microecological niches. It suggests that the gene expression and protein synthesis of *Bacillus* species and related *Staphylococcus aureus* may change under the influence of different carbon sources, thus affecting its ability to repress bacteria.

Against this background, an important question is whether cellobiose and microcrystalline cellulose affect the cellular microenvironment and some functions of *B. licheniformis NXU98*, which is the focus of our study. In this study, we isolated a strain of *Bacillus licheniformis* from the rumen of Holstein dairy cows and named it *NXU98*. During the culture process, we observed that different carbon sources had varying effects on its growth. Hence, we hypothesized that it might be possible to influence its biological function through different carbon sources during its growth. Combined transcriptome and proteome analysis were utilized to study the gene expression and protein synthesis of *B. licheniformis* under the influence of different carbon sources in order to better understand the mechanism of its effect on the regulation of the microenvironment.

Transcriptome is a technical tool adopted to study the expression of all genes in cells, which can be used to analyze the differences in gene expression levels between different samples using RNA sequencing and thus uncover the key genes (Lowe et al., 2017). Proteomics is a technical tool employed to study the composition and function of proteins that can be identified in different samples by mass spectrometry to analyze the protein composition of *B. licheniformis* under different carbon sources and its effect on bacterial inhibition (Shiny et al., 2020).

Through these analytical tools, the differences in gene expression and protein synthesis of *B. licheniformis NXU98* under the influence of different carbon sources can be better explored to further reveal its mechanism and influence on bacterial inhibition, thus providing a more in-depth theoretical support for future research and application of probiotics.

## 2. Materials and methods

### 2.1. Rumen fluid collection

Animals used in this experiment were cared for and maintained in accordance with a protocol approved by the Chinese Standards for the Use and Care of Research Animals. To obtain *B. licheniformis NXU98* from rumen fluid, samples were collected from three healthy Holstein cows. Then, 50 mL of rumen fluid was collected via the ruminal fistula in the ventral rumen sac and placed in sterile centrifuge tubes. Further, the rumen fluid sample was mixed and filtered through four layers of gauze and stored at 4°C for further analysis.

### 2.2. Isolation of bacterial strain

The isolation and purification of strains have been described in a previous study by Zhang et al. (2018). Briefly, approximately 1 mL of the rumen liquid was mixed with 9 mL of enrichment medium (cellobiose 1.0 g, K_2_HPO_4_·3H_2_O 0.5 g, MgSO_4_·7H_2_O 0.5 g, MnSO_4_ 0.025 g, yeast extract 5.0 g, and peptone 5.0 g mixed with distilled water to make up 500 mL, sterilized, and stored at 4°C) and then cultured at 39°C for 24 h.

To observe the colony characteristics of the isolated strain, the dilution plate method was used to obtain single colonies. An isolation medium was prepared to isolate the strain (CMC-Na 2.5 g, K_2_HPO_4_·3H_2_O 0.5 g, MgSO_4_ 0.25 g, NaNO_3_ 1.5 g, and ager 8.5 g, and mixed with distilled water to make up 500 mL). The strains cultured in the enrichment medium were inoculated on the isolation medium at 39°C for 48 h. Then, the Congo red stain was used to stain the strain, and a transparent circle strain was chosen to be inoculated on an isolation medium again until the gram stain and shape were stable.

The isolated and screened bacilli licheniformis derived from the rumen were inoculated on a liquid medium with cellobiose and microcrystalline cellulose as the only carbon source, respectively, and cultured at 39°C for 8 h. Then, transcriptomic and proteomic analysis were performed.

### 2.3. Identification of bacterial strain

#### 2.3.1. Gram’s stain

Creamy-white colonies were selected with rough surfaces and wrinkled edges for further culturing. After culture, single colonies were selected for Gram staining. Gram-positive colonies were selected for purification culturing until a pure stain was achieved.

#### 2.3.2. 16S rDNA sequences

The genomic DNA of the newly identified purification strain was extracted for the amplification of the 16S rDNA with two bacterial universal primers: 7F: 5′-CAGAGTTTGATCCTGGCT-3′, and 1540R: 5′-AGGAGGTGATCCAGCCGCA-3′. The extracted DNA was sent to Shanghai Sangon Biotechnology for high-throughput sequencing. The 16S rDNA sequences were compared with the ribosomal database,^1^ and the bacteria were identified at the species level of phylum, family, and genus. The strain’s gene sequence homology was verified through prokaryotic transcriptomics (Shanghai Bipu Biotechnology Co., Ltd., Shanghai).

### 2.4. RNA preparation for sequencing

RNA samples were isolated from the bacteria and were only supplied with cellobiose or microcrystalline cellulose as a carbon source and incubated at 39°C and 180 r/min for 8 h (OD600 = 0.5–0.8). Cells were collected by centrifugation at 12,000 rpm for 5 min and then transferred to a 10 mL centrifuge tube after grinding in liquid nitrogen.

Cells were then lysed in 1 mL of TRIzol for 30–60 s. To the lysate, 200 μL of chloroform was added, and the sample was then mixed by inversion and incubated at room temperature for 15 min. The sample was then centrifuged at 12,000 rpm for 15 min at 4°C, and the supernatant was precipitated with an equal volume of isopropanol at room temperature for 10 min. After centrifugation, the supernatant was discarded, and the pellet was air-dried and dissolved in 20–40 μL of RNase-free water. Total RNA was treated with RNase-free DNase I for 30 min at 37°C to remove genomic DNA. Total RNA was extracted using the Trizol reagent from the bacteria. The Nanodrop 2000 was used to detect the concentration and quality of extracted RNA. The integrity of the RNA was detected by agarose gel electrophoresis, and the RIN value was determined by Agilent 2100.

One milligram of RNA from each sample was used for further analysis. Library construction was generated using the Illumina Hiseq Stranded mRNA Library Preparation Kit TruSeq (Illumina, San Diego, CA, United States) and then sequenced on a NovaSeq 6000 instrument at Macrogen, Inc. (Macrogen, Seoul, Republic of Korea) to obtain approximately 100 million 2 × 150 bp reads/sample.

### 2.5. Transcriptome analysis

The quality of raw reads was assessed with FastQC 0.11.4 tools. The FastqStat tool was used to trim the sequence reads by removing the remaining Illumina Hiseq adaptors in the reads. This program also discards unpaired reads from paired-end RNA-seq output. After removing low-quality reads, the read quality was assessed again using FastQC. Forward and reverse reads were mapped to the *B. licheniformis NXU98* draft genome (Esmaeilishirazifard et al., 2017) to remove the non-target reads using the HISAT 2 2.1.0 tool with a minimum fragment length of 20. The DESeq2 tool was used to analyze differentially expressed genes between the two groups. Before performing differential gene expression analysis for each sequencing library, a proportional normalization factor was used to adjust the read count through the edgeR package. The *p*-value was adjusted using the Benjamin and Hochberg method.

### 2.6. Protein extraction and HPLC-MS/MS analysis

For each sample, 300 μL SDT was added, and the sample was kept in a boiling water bath for 5 min, treated with ultrasound for 2 min, then immersed again in a boiling water bath for 5 min, and the supernatant was obtained by centrifugation at 4°C and 20,000 g.

FASP enzymolysis was performed with 300 ug of protein in each sample. The steps were as follows: dithiothreitol (DTT) was added to 100 mM in each sample, kept in a boiling water bath for 5 min, and then cooled to room temperature. Then, 200 μL UA of buffer (8 M Urea, 150 mM Tris-HCl, pH 8.0) was added, mixed well, transferred into a 10 KD ultrafiltration centrifuge tube, and centrifuged at 12,000 g for 15 min. To this, 200 μL of UA buffer was added, centrifuged at 12,000 g for 15 min, and the filtrate was discarded. Further, 100 μL of IAA (50 mM IAA in UA) was added at 600 rpm. The mixture was oscillated for 1 min, avoided light at room temperature for 30 min, and centrifuged for 12,000 g for 10 min. To this, 100 μL of UA buffer was added, centrifuged at 12,000 g for 10 min, and repeated twice, following which 100 μL of NH_4_HCO_3_ buffer was added, centrifuged at 14,000 g for 10 min, and repeated twice. Further, 40 μg of Trypsin buffer (6 μg Trypsin in 40 μL NH_4_HCO_3_ buffer) was added at 600 RPM for 1 min at 37°C for 16–18 h. A new collection tube was replaced, centrifuged for 12,000 g for 10 min, and the filtrate was collected and added with an appropriate amount of 0.1% TFA solution. After enzymatic hydrolysis, the peptide segment was desalted using a C18 cartridge and lyophilized under vacuum. After drying, the peptide was redissolved in 0.1% formic acid, and the concentration of the peptide was determined for LC-MS analysis (Schwanhausser et al., 2013).

Appropriate peptide segments were taken from each sample for chromatographic separation using the nanoliter flow rate Easy nLC 1200 chromatography system (Thermo Fisher Scientific, USA). To prepare the buffer, 0.1% formic acid aqueous solution (liquid A) and 0.1% formic acid acetonitrile aqueous solution (acetonitrile: 85%) (liquid B) were prepared. The column was balanced with 95% liquid A. The samples were injected into a trap column (100 μm × 20 mm, 5 μm, C18, Dr. Maisch GmbH) and then subjected to a chromatographic column (75 μm × 150 mm, 3 μm, C18, Dr. Maisch GmbH) for gradient separation. The flow rate was 300 nL/min. The liquid phase gradient was set as follows: from 0 min to 2 min, the linear gradient of liquid B ranged 5%–8%; from 2 to 90 min, the linear gradient of liquid B ranged 8%–23%; from 90 to 100 min, the linear gradient of liquid B ranged 23%–40%; and from 100–108 min, the linear gradient of liquid B ranged 40%–100%. From 108 to 120 min, liquid B was maintained at 100%.

The peptide was isolated and then analyzed by DDA (data-dependent acquisition) mass spectrometry using a Q-Exactive HF-X mass spectrometer (Thermo Scientific). The duration of analysis was 120 min, and the detection mode was positive ion with a parent ion scanning range of 300–1,800 *m*/*z*. The primary mass spectrometry resolution was 60,000@*m*/*z* 200, with an AGC target of 3e6 and a primary maximum IT set at 50 ms. After each full scan, secondary mass spectrometry (MS2 scan) of the 20 highest-intensity parent ions was taken. The secondary mass spectrometry resolution was set at 15,000@*m*/*z* 200, with an AGC target of 1e5 and level 2 maximum IT of 50 ms. The MS2 activation type was HCD, the isolation window was 1.6 *m*/*z*, and the normalized collision energy was 28 (Shu et al., 2018).

### 2.7. Relational analysis of proteome with transcriptome

Protein Pilot software (version 5.0) (AB SCIEX) was used to match the mass spectrometry raw data to the contents of a *B. licheniformis* database with the Paragon algorithm (Shilov et al., 2007). The differentially expressed proteins were identified based on a fold change of >1.2 or <0.8 and a *p*-value of <0.05. A comparative analysis between proteome and transcriptome was carried out on the online platform of Majorbio Cloud Platform,^2^ and the heat map was generated using Heatmapper.^3^ The Kyoto Encyclopedia of Genes and Genomes (KEGG) pathways enriched among the differentially expressed protein and differentially expressed genes were also identified.

### 2.8. MIC assay

*B. licheniformis NXU 98* was cultured for 72 h and centrifuged at 12,000 r/min at 4°C for 10 min. A 0.1 mL sample of the supernatant was then added to a sterile 96-well plate and diluted by the two-fold method. 0.1 mL of an approximately 10^6^ CFU/mL bacterial suspension was added to obtain concentrations of 0.1, 0.2, 0.4, 0.78, 1.56, 3.12, 6.25, 12.5, 25, and 50%, and the mixture was incubated at 37°C for 18 h. The absorbance value was read by SuPerMax 3000AL (Shanpu, Shanghai, China).

### 2.9. Statistical analysis

#### 2.9.1. Statistics and analysis of transcriptomic data

The original sequencing data was filtered, and the data containing sequencing connector sequences, low-quality read segments, high n-rate sequences, and excessively short sequences were excluded. At the same time, Q20, Q30, and GC contents were calculated to obtain high-quality sequencing data. The sequencing data was compared with the reference genome by Hisat2 (parameter: -p 10-rna-strandness R), and the compared data was used for subsequent analysis. Pearson’s correlation coefficient (*r*) was used as an evaluation index of biological repeat correlation. The closer *r*^2^ is to 1, the stronger the correlation between the two duplicate samples. The heat map was drawn by plot_cor_exp (v1.1.0). DESeq2 software (1.16.1) was used for differential expression gene analysis between the two groups. Prior to the differential gene expression analysis, the read count was adjusted via the edgeR package for each sequenced library by a proportional normalization factor. The *p*-values were adjusted using the Benjamin and Hochberg method. The corrected *p*-values and |log_2_FC| <1 and FDR <0.05 were used as the threshold for significant differential expression. Using the GO database, go_anot_exp, version v 1.4.0, genes were classified according to their involvement in biological processes, cellular components, and molecular functions. Using the KEGG database, kegg_anot_exp, version v 1.4.0, the genes were classified according to the participating pathway. All genes were used as the background list, while differential genes were used as the candidate list screened from the background list, and finally, Fisher’s exact test was used. To control the calculated false positive rate, four multiple-test methods (Bonferroni, Holm, Sidak, and false discovery rate) were used to correct the *p*-value. Under normal circumstances, when the corrected *p*-value is ≤0.05, it is believed that there is a significant enrichment of GO function or KEGG pathway.

#### 2.9.2. Statistics and analysis of proteomic data

We used MaxQuant (V1.6.0.16) to analyze MS data. At the matching level of peptide profiles and protein levels, the matching level was <1% for error finding rate (FDR) filters and export database search results. Marker-free quantization was performed in MaxQuant using intensity determination and normalization algorithms (Luber et al., 2010; Tampe and Zeisberg, 2013; Cox et al., 2014). The mass spectrometry proteomics data have been deposited to the ProteomeXchange Consortium via the PRIDE (Perez et al., 2022) partner repository with the dataset identifier PXD042310. The LFQ intensity of each protein in different samples was calculated, and quantitative protein ratios were weighted and normalized using the median in MaxQuant software. Fold change ≥1.5 times and *p*-value <0.05 protein were taken as the values for the significantly differentially expressed protein.

The bioinformatics data were analyzed using Perseus (Tyanova et al., 2016), Microsoft Excel, and R statistical computing software. The pheatmap software package based on the open-source statistical language R25 was used to perform hierarchical clustering analysis with Euclidean distance as the distance metric. The KEGG enrichment analysis was performed by Fisher precision test, and FDR correction was performed for multiple tests. The construction of the protein–protein interaction (PPI) network was also carried out using the string database of Cytoscape software (Kohl et al., 2011).

## 3. Results

### 3.1. Screening and identification of strains

**Figure 1:**
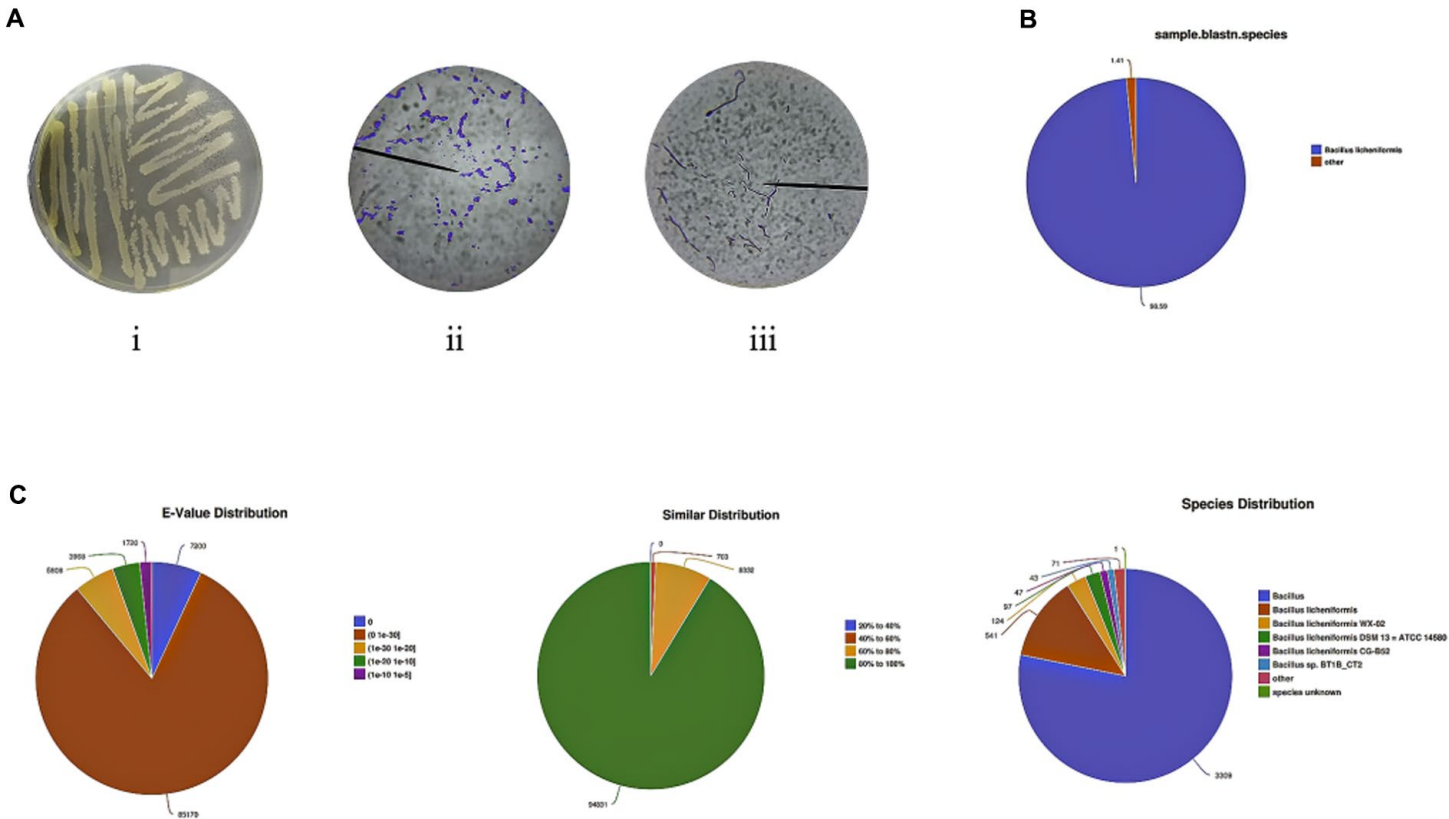
Isolation and identification of new *Bacillus licheniformis* from rumen fluid. **(A)** Screening of *B. licheniformis* and results of Gram staining. **(B)** Species comparison of screening strains. **(C)** Distribution of NR library comparison results.

After 24 h of incubation, the bacterial solution was shaken evenly, and the inoculum was diluted into the isolation medium by scribing with a disposable sterile inoculation loop, sealed with a sealing film, and incubated at 39°C for 48 h. The initial screening of the resulting strain showed a beige colony with a rough and wrinkled surface and an untidy edge. The edge of the colony was hairy and deepened in color after prolonged incubation, and the surface of the colony had a sticky liquid (Figure 1A). The bacterial colonies were then stained with Congo red, and those with hyaline circles were identified for purification. As shown in Figure 1A (i and ii), the strains obtained by screening were morphologically observed by Gram staining, and the strains had long rod-shaped, single, paired, or chain-arranged Gram-positive bacilli.

The strain obtained from this study was 99% similar to the 16S ribosomal RNA gene of *B. licheniformis* strains CCMMB 933 and 810 and 100% similar to the 16S ribosomal RNA gene of *B. licheniformis* strains CCMMB 928, 925, and 885 (Supplementary Figure S1). According to the requirement of the 16S region of the ribosome gene in bacterial identification, greater than 99% can be regarded as the same species; therefore, this strain can be identified as *B. licheniformis*.

The prokaryotic transcriptome sequencing gene sequences of the identified strains were compared with *B. licheniformis* using the NCBI gene database, and the gene similarity was 98.59%. In addition, it was found that the identified *B. licheniformis* belonged to the genus *Bacillus* and the species *B. licheniformis*, which had high similarity with three subspecies of *B. licheniformis WX-02*, *B. licheniformis DSM 13 = ATCC 14580*, and *B. licheniformis CG-B52* (Figures 1B,C). This indicates that the strain obtained in this study is a subspecies of *B. licheniformis*, but there is no exact sequence match with it in the NR database. Therefore, our laboratory named the strain *B. licheniformis NXU98* (GenBank: OR001969).

### 3.2. Antibacterial activity

As shown in Table 1; Supplementary Table S3, in the MC group, the proportion of supernatants greater than 0.2% could express an antibacterial effect on *E. coli*, and supernatants more than 0.8% had an antibacterial effect on *S. aureus*. In the CB group, when the proportion of supernatant was more than 1.6%, it showed a significant antibacterial effect on *E. coli*. The antibacterial effect was more obvious with the increase in the concentration. However, there was no significant effect on *S. aureus.*

**Table 1:** *Bacillus licheniformis NXU98* against different bacteria.

Item	Minimum inhibitory concentration of *NXU98* (%)											SEM	*p*-value
	0.0	0.1	0.2	0.4	0.8	1.6	3.1	6.3	12.5	25.0	50.0		
**MC**													
*E. coli*	0.925^A^	0.879^A^	0.822^B^	0.799^BC^	0.753^CD^	0.736^D^	0.750^D^	0.723^D^	0.728^D^	0.717^D^	0.727^D^	0.011	<0.001
*S. aureus*	0.889^AB^	0.907^A^	0.854^ABC^	0.852^ABCD^	0.823B^CD^	0.810^CD^	0.810^CD^	0.814^CD^	0.777^D^	0.786^CD^	0.801^CD^	0.016	0.008
**CB**													
*E. coli*	0.967^A^	0.941^A^	0.950^A^	0.867^A^	0.807^A^	0.799^AB^	0.802^AB^	0.783^B^	0.767^B^	0.768^B^	0.756^B^	0.009	<0.001
*S. aureus*	0.930	0.924	0.913	0.905	0.896	0.900	0.890	0.889	0.893	0.867	0.801	0.026	0.983

^A,B,C^Means within the same row with no common superscript differ significantly (*p* < 0.05).

### 3.3. RNA-Seq data analysis

Transcriptomic analysis was performed to explore the transcription levels of related genes involved in several pathways. The base-pair GC content distribution and the sequencing error distribution rate (Q30) were used to evaluate the quality of the sequencing data to ensure the accuracy of the test results. A total of 12 GB (each group was greater than 6 GB) of clean reads and 6,344 unigenes were detected from the Illumina high-throughput sequencing platform. The Q20 and Q30 of each sample were over 98.61% and 95.95%, indicating that the data quality satisfied the analysis requirements. In addition, the base-pair GC and AT contents of each sequencing read should be equal and stable throughout the sequencing process according to the principles of random interruption of sequences and double-strand complementarity. As shown in Supplementary Table S1, the GC content of six samples was stable at 51%. These results indicated that the sequencing results were good in quality and acceptable for the next analysis.

**Figure 2:**
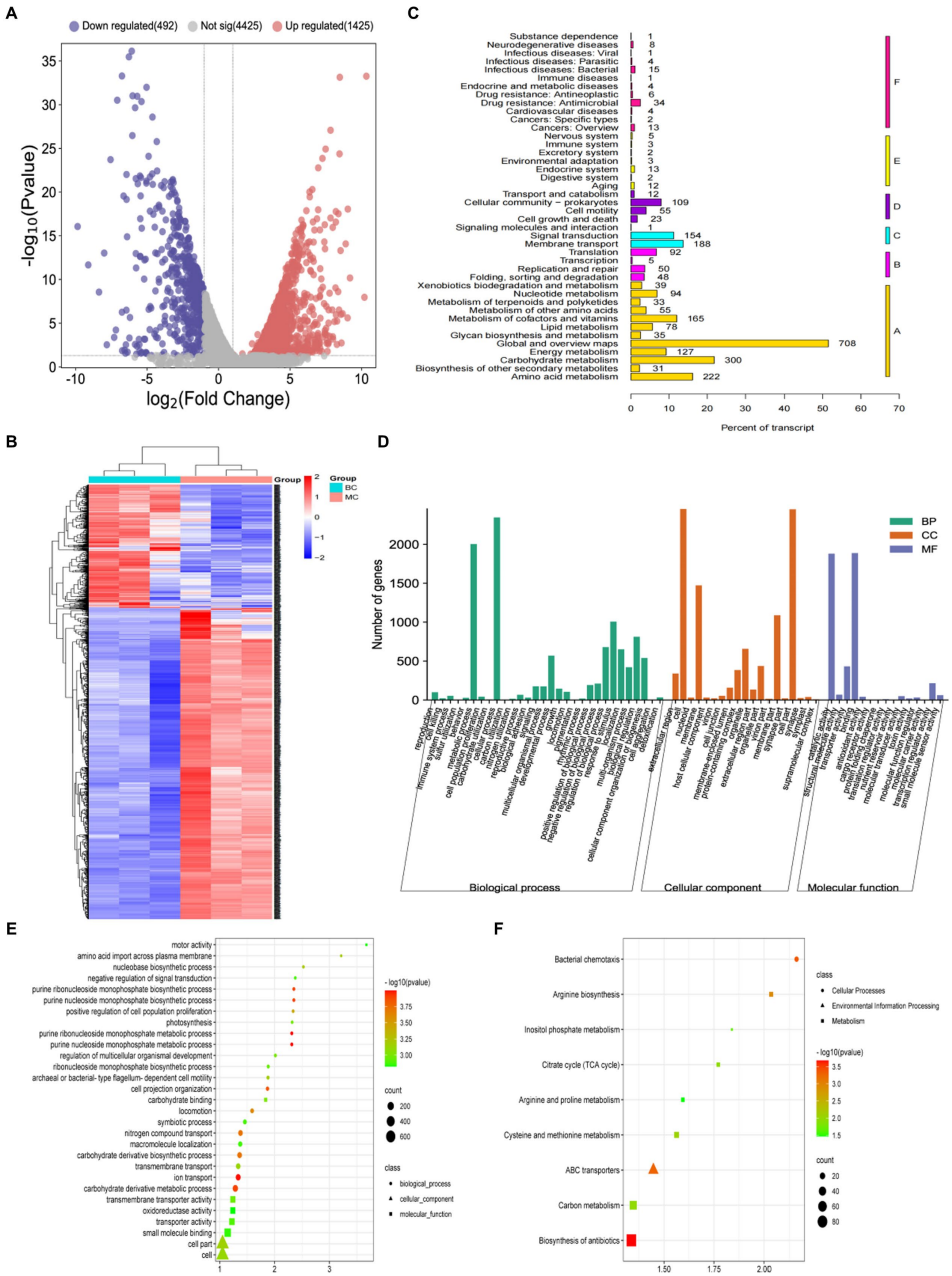
**(A)** A volcano map of DEGs changed by different carbon sources. **(B)** Heatmap of the transcriptome of *B. licheniformis NXU98* with different carbon source **(C)** KEGG metabolic pathway classification of transcripts. The ordinate is the name of the KEGG metabolic pathway, and the abscissa is the number of genes annotated to the pathway and the proportion of the number to the total number of genes annotated. **(D)** GO analysis of DEGs. **(E)** Scatter plot of GO functional enrichment of DEGs. **(F)** Scatter plot of KEGG functional enrichment of DEGs.

Diversity and similarity analyses within and between groups were performed before comparing differentially expressed genes (DEGs) between the MC and CB groups. The Pearson correlation coefficient analysis showed that the intra-group R2 was between 0.75–0.84 for the MC group and 0.74–0.87 for the CB group, while the inter-group R2 between MC and CB groups was between 0.46–0.63. This indicated that the sequencing data of the intra-group samples had high similarity and significant biological reproducibility, while the inter-group correlation was low, and there were significant differences (S2). Meanwhile, principal component analysis was used to evaluate intergroup differences and intragroup replications. All these results indicated that different carbon sources had significant regulatory effects on gene expression in *B. licheniformis NXU98*. With three replicates per group, there were 1,665 significant DEGs, 1,192 significantly upregulated DEGs, and 473 significantly downregulated DEGs in the MC group compared to the CB group (corrected *p*-values and |log_2_FC| >1 and FDR <0.05), including 586 newly predicted genes and 822 unknown genes (Figures 2A,B).

### 3.4. Bioinformatic analysis of differentially expressed genes

To further understand the antimicrobial effect of DEGs with different carbon sources on *B. licheniformis*, GO and KEGG enrichment were used to analyze all DEGs. The (S3) results of GO enrichment showed that the total number of genes and significantly differentially expressed genes between different carbon sources were 3,204 and 872, respectively. According to the GO analysis, 32 biological processes, 19 cellular components, and 15 molecular functions were enriched (Figure 2C). The top five terms annotated in the biological process (BP) category were localization, ribose phosphate metabolic process, ribonucleotide metabolic process, purine ribonucleotide metabolic process, and purine nucleotide metabolic process (Figure 2E). In the cellular component (CC), DEGs were enriched in bacterial-type flagellum, bacterial-type flagellum basal body, bacterial-type flagellum part, cell part, cell projection part, organelle part, cell, plasma membrane, and membrane (Figure 2E). In addition, Figure 2E shows that carbohydrate binding, transmembrane, transporter activity, and active ion transmembrane transporter activity were enriched in molecular functions (MF).

KEGG pathway enrichment (Figure 2D) revealed that, in *B. licheniformis* cultured with different carbon sources, the DEGs were involved in 139 KEGG metabolic pathways. The metabolic pathways with a high distribution of DEGs were mainly involved in the biosynthesis of secondary metabolites, microbial metabolism in different environments, antibiotic biosynthesis, two-component systems, ABC transporter proteins, amino acid biosynthesis, carbon metabolism, and population sensing. Among them, the top five significantly enriched (*p*-value FDR ≤0.05) KEGG metabolic pathways were flagellar assembly, biosynthesis of antibiotics, bacterial chemotaxis, ABC transporters, and arginine biosynthesis (Figures 2E,F). These results suggest that the addition of different carbon sources to the substrate can modulate the physiological and biochemical processes within *B. licheniformis NXU98*, especially the biosynthesis of secondary metabolites and the carbon metabolism to accelerate cell growth, bacterial chemotaxis, and antimicrobial biosynthesis.

### 3.5. Proteomics annotation analysis

From the quantitative results in S4, it was known that the protein concentrations in the samples were in the range of 0.5–1.5 μg/μL, and the total amount met the needs of LC-MS/MS analysis. Approximately 15 μg of protein was taken from each sample for SDS-PAGE. The gel electrophoresis results showed normal protein extraction in each sample, and the protein electrophoresis bands showed good parallelism within and between sample groups (MC group 1-1, 1-2, and 1-3, CB group 2-1, 2-2, and 2-3). By searching and analyzing the proteins in the total proteomic library of *B. licheniformis NXU98* after changing the carbon source, a total of 1,918 proteins were identified in the MC group and 1,830 in the CB group. The Veen map showed (S5) that the number of protein groups specific to the MC and CB groups was 115 and 27, and there were no differences in the 1803 group of proteins.

**Figure 3:**
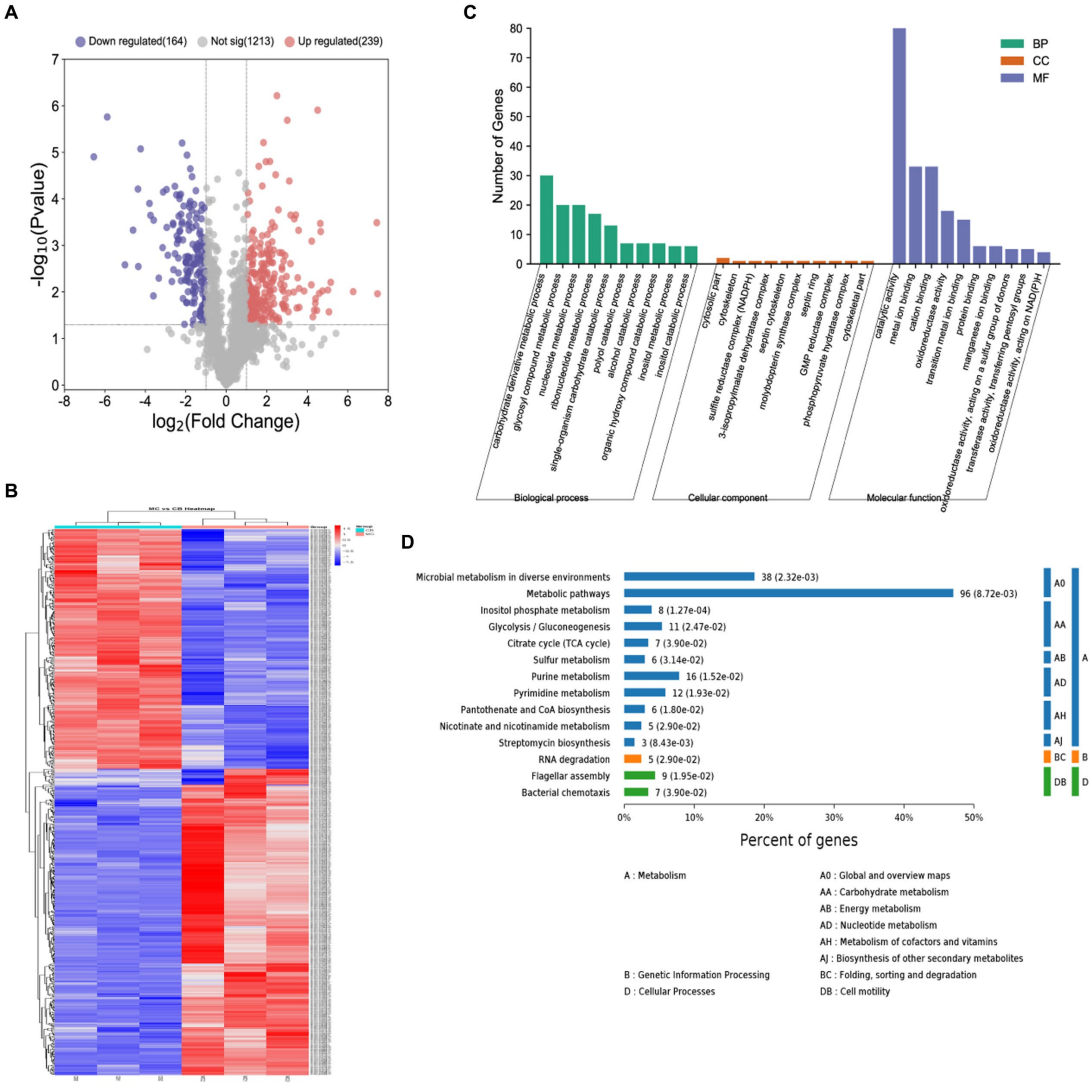
Proteomics analysis of *B. licheniformis NXU98* with different carbon sources. **(A)** A volcano map of DEPs changed by different carbon sources. **(B)** Heatmap of the proteomics of *B. licheniformis NXU98* with a different carbon source **(C)** GO analysis with significant differences in the proteome. **(D)** KEGG enrichment analysis of different proteins.

The volcano map of differentially expression proteins (DEPs) showed (Figure 3A) that a total of 729 DEPs were identified by *B. licheniformis NXU98* under the different carbon sources treatment. Among the DEPs, 327 were significantly upregulated, and 256 were significantly downregulated (Figure 3A). A heat map (Figure 3B) generated from the normalized expression of aligned reads of the MC and BC groups revealed hierarchical clustering of all the differentially expressed transcripts with a *p*-value of <0.05. The GO analysis showed that the significant DEPs were enriched in single-organism carbohydrate catabolic process, glycosyl compound metabolic process, nucleoside metabolic process, polyol catabolic process, alcohol catabolic process, and other BPs (Figure 3C). In MF, the DEPs were mainly enriched in oxidoreductase activity, acting on a sulfur group of donors, oxidoreductase activity, catalytic activity, metal ion binding, and in the CC category (Figure 3C). In CC, there were no significant proteins changed in GO enrichment.

The KEGG pathway analysis revealed (Figure 3D) that the two groups of differential proteins were annotated to a total of 80 metabolic pathways. The significantly enriched metabolic pathways were inositol phosphate metabolism, microbial metabolism in diverse environments, β-lactam resistance, streptomycin biosynthesis, metabolic pathways, purine metabolism, pantothenate and CoA biosynthesis, pyrimidine metabolism, flagellar assembly, glycolysis/gluconeogenesis, nicotinate and nicotinamide metabolism, RNA degradation, sulfur metabolism, citrate cycle (TCA cycle), bacterial chemistry cycle, and bacterial chemotaxis.

### 3.6. Analysis of the association between the proteomic and transcriptomic data

**Figure 4:**
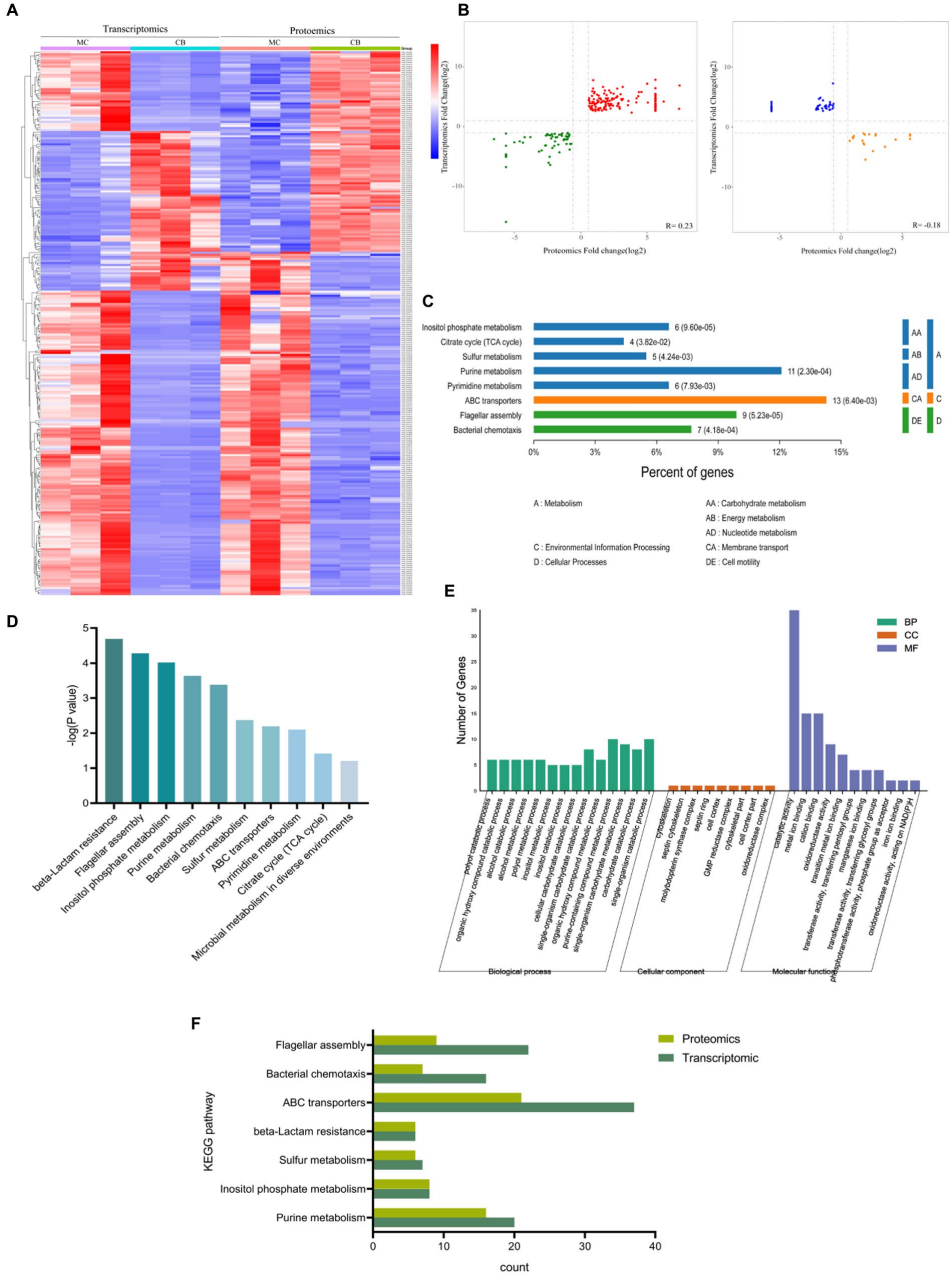
Combination of transcriptomic and proteomics analysis with different carbon sources. **(A)** Heatmap of transcriptomic and proteomics. **(B)** Correlation analysis of differentially expressed proteins and genes with the same and opposite tendency of change. **(C,D)** KEGG enrichment analysis of comparator group. **(E)** GO enrichment and *p*-value analysis of comparator group. **(F)** KEGG analysis of genes with significant differences in the proteome and transcriptome.

To combinate transcriptional and protein expression data, DEPs from the proteomic analysis were compared with annotated RNA-seq libraries. The results showed that all 1,943 proteins had the same expression pattern as their mRNAs and that the expression of all proteins was directly regulated at the transcriptional level (S6). The clustering analysis of these 326 DEGs and DEPs (Figure 4A) showed that DEGs and DEPs were equally reliable for further analysis. Figure 4B shows that a total of 266 genes were significantly different between transcript level and protein level. Among these, there were 208 differential genes with the same trend and 58 differential genes with the opposite trend. This indicated that most of the genes’ proteome and transcriptome results corroborated each other. However, there may be some differential genes that undergo mRNA post-transcriptional regulatory mechanisms and protein post-translational modifications.

The KEGG annotation results showed (Figure 4C) that the common significantly different genes were mainly located in biological metabolic processes such as alcohols, hydroxyl compounds, purines, and carbohydrates. The results of the KEGG functional annotation (Figure 4D) showed that the common pathways of transcriptome and proteome under different carbon source conditions were β-lactam resistance, flagellar assembly, inositol phosphate metabolism, purine metabolism, bacterial chemotaxis, sulfur metabolism, and ABC transporters. The GO analysis revealed that 443 BP, 30 CC, and 161 MF relative pathways were enriched in a combination of transcriptome and proteome (the significant pathways are shown in Figure 4E). Further analysis of transcriptome and proteome significant KEGG signaling pathways (*p* < 0.05) showed that the total KEGG metabolic pathway was mainly involved in the following seven metabolic pathways: purine metabolism, inositol phosphate metabolism, sulfur metabolism, ABC transporter protein, bacterial chemotaxis, and flagellar assembly (Figure 4F).

## 4. Discussion

*B. licheniformis* is a commonly used and generally considered safe probiotic species (Maki et al., 2019). The main sources of *B. licheniformis* are soil, milk, animal feces, and food (Elke and Paul, 2004). In this study, we first obtained rumen fluid from the rumen of healthy Holstein cows. The strain was identified and characterized as a new sub-population *B. licheniformis NXU98* by combining classical culture identification and molecular biology. The gene sequences of the prokaryotic transcriptome of the strain were compared with *B. licheniformis* genes by the NCBI gene database, and the gene similarity was 98.59%. Previous studies have shown that *B. licheniformis MCC2514* has more genes encoding hydrolytic sugars and thus has more ability to degrade polysaccharides (Dindhoria et al., 2022). Further, our results of homologous genes showed that they had a variety of sugar catabolisms that can secrete a variety of carbohydrases.

Ghali et al. (2017) demonstrated that *Fibrobacter succinogenes S85* exhibited rapid growth on cellobiose and showed aerobic growth on lactose medium containing 0.05%–0.2% cellobiose while inhibiting the growth of lactose-degrading β-glucosidase. Shulami et al. (2020) found that adding cellobiose to the culture medium increased the gene expression levels of *celA*, *celB*, *celC*, *celD*, and *celR* of *B. thermoliboria* by 7–15 times compared with normal carbon sources and regulated the expression of galactose ABC transporter. Riederer et al. (2010) found that, when cellobiose was used as a carbon source, it can significantly increase the levels of cellulosome enzymes, intracellular metabolic enzymes, transcriptional regulators, sigma factors, signal transducers, transporters, and hypothetical proteins. Additionally, microcrystalline cellulose as a carbon source can enhance lignin dissolution activity (Osiro et al., 2017). Furthermore, under medium temperature conditions, *Clostridium populeti FZ10* can directly convert lignocellulose into biohydrogen using isolated microcrystalline cellulose as a substrate. In addition, the microcrystalline cellulose in the deep-rooted CQDs (CQDs-HT) showed excellent bactericidal potential (Mogharbel et al., 2023).

To better explain the potential regulatory mechanisms of *B. licheniformis NXU 98* in cellobiose and microcrystalline cellulose, an integrated transcriptomic and proteomic analysis was conducted to explore the transcription levels of related genes involved in bacterial inhibition. The results of the GO enrichment and KEGG ortholog analyses of genetic transcription indicated that 5 KEGG metabolic pathways were significantly enriched, including flagellar assembly, biosynthesis of antibiotics, bacterial chemotaxis, ABC transporter, and arginine biosynthesis. The *B. licheniformis NXU98* isolated in this study has the ability to regulate ecological niches, which are regulated by different carbon sources.

### 4.1. Effects of different carbon sources on antibacterial peptides of *Bacillus licheniformis NXU98*

Previous studies have shown that the production of antimicrobial peptides was regulated by their own synthetic genes and by transport proteins on the cell membrane surface, among which the ABC transporter family plays a key role in the transport of antimicrobial peptides (Gebhard, 2012; Beis and Rebuffat, 2019; Ahmad et al., 2020). An analysis of transcriptomics combined with proteomics showed significant activation of ABC transporter family proteins under MC group treatment conditions. These include several core components associated with oligopeptide transport, including *OPPA*, *OPPB*, *OPPC*, *OPPD*, and *OPPF*. The effect of the carbon source has been reported to interfere with antibiotic synthesis (Sánchez et al., 2010). In this study, we also found that MC, relative to BC biosynthesis of antibiotic signaling pathways, was enriched in KEGG in transcriptome sequencing. Further analysis of the biosynthesis of antibiotic signaling pathways revealed that several of the antibiotic synthesis signals were activated, including purine metabolism, novobiocin biosynthesis, monobactam biosynthesis, puromycin biosynthesis, and phenazine biosynthesis. These results further suggest that MC, as a distinct carbon source, can increase the bacterial inhibitory capacity of *B. licheniformis NXU98* by enhancing the metabolic synthesis of antibiotics and improving the efficiency of antimicrobial peptide transport. These results suggest that this strain has the potential to improve microenvironmental regulation of the internal flora homeostasis of the surrounding environment and that this ability is regulated by different carbon sources. Therefore, we conducted antibacterial tests using different carbon source culture supernatants extracted from *B. licheniformis NXU98* to validate the results of transcriptome and proteome analysis. The results of the antibacterial tests showed that the MC and CB groups significantly inhibited the growth of *E. coli*, and a higher proportion of supernatant resulted in a more pronounced antibacterial effect. However, for *S. aureus*, the CB group did not show significant inhibition, which may be due to the inhibitory effect on some ABC transporter protein family gene expression caused by cellobiose, resulting in decreased antibacterial activity against Gram-positive bacteria. The specific mechanism needs further investigation.

### 4.2. Effects of different carbon sources on bacterial chemotaxis of *Bacillus licheniformis NXU98*

Apart from the ability to inhibit bacteria, we found that other biological behaviors of *B. licheniformis NXU98* were modulated by different carbon sources. Bacterial chemotaxis is when bacteria change their direction of movement in response to changes in chemical concentration in the environment. Chemotaxis is one of the most important ways in which bacteria interact with their environment and can influence their growth, metabolism, signaling, gene expression, and community behavior (Wang and Ford, 2010; Keegstra et al., 2022). Combined analysis of transcriptomics and proteomics showed that MC was significantly upregulated relative to genes associated with CB chemotaxis and flagellar assembly. KEGG pathway annotation revealed 16 significantly different genes annotated to the bacterial chemotaxis pathway, including *cheA*, *cheW*, *cheY*, *cheB*, *cheD*, *cheV*, *cheC*, *FliG*/*M*/*NY*, *MotA*, and *MotB*. These series of genes can form a complete bacterial chemotaxis pathway and lead to enhanced chemotaxis of *B. licheniformis NXU98*. Besides, a series of genes related to flagellar assembly synthesis, including the *FliH* and *FlgB* gene family, were also significantly higher under MC treatment. Similarly, Liu et al. (2018) reported that pseudomonas proteins H78 can regulate flagellar assembly by modulating *FlgF* and *FlgB* at different carbon sources. *B. licheniformis NXU98* detects chemical gradients through receptors and changes their movement patterns by regulating the rotation of flagella. This behavior allows bacteria to seek out beneficial nutrients or avoid harmful toxins. It further regulates the distribution of nutrients and waste products in the microenvironment, thereby influencing the growth and metabolism of bacteria in the micro-environment.

### 4.3. Effect of *Bacillus licheniformis NXU98* with different carbon sources on rumen microecological environment

Lactic acid is one of the main products of rumen microbial fermentation, which can provide energy and carbon sources and promote the proliferation and activity of rumen microorganisms. Excess lactic acid lowers the pH of the rumen and inhibits the growth of other beneficial microorganisms, leading to rumen dysfunction (Fu et al., 2022). In the present study, MC was significantly downregulated relative to CB differentially expressed proteins Eno, Gpml, PgcA, PgK, PgcA, and PfkA expression, leading to reduced pyruvate synthesis in the glycolytic pathway and consequently reduced LDH expression, leading to reduced lactate synthesis. Therefore, the glycolysis of *B. licheniformis NXU98* in this study was affected by different carbon sources, and MC treatment can inhibit the glycolytic process and reduce the production of lactic acid. Cellulase plays a key role in the digestion of plants in ruminants. Bacteria in the rumen secrete cellulases that help ruminants convert cellulose into energy and nutrients (Miao et al., 2019). Compared to CB, the protein expression of AcoC, AcsA, MdH, MmgD, SucC, SucD, SdhB, and PckA of *B. licheniformis NXU98* was significantly higher under MC treatment. The results suggest that the MC, when used as a carbon source, is involved in the regulation of gluconeogenesis and the TCA cycle, synthesizing high levels of acetyl coenzyme A, further producing oxaloacetate and generating more cellulase through the gluconeogenic pathway. The above experimental results suggest that the strain of *B. licheniformis NXU98* identified in this study has potential application in regulating the microecological environment of ruminant gastric juice.

## 5. Conclusion

In our study, we isolated and identified a new strain of *B. licheniformis NXU98* from bovine rumen fluid. Further, we analyzed the response of the *B. licheniformis NXU98* strain to two distinct carbon sources through a combination of transcription and protease assays. When microcrystalline cellulose was used as the sole carbon source, *B. licheniformis NXU98* could inhibit bacterial activity by ABC transporter protein and antimicrobial peptide synthesis. Additionally, *B. licheniformis NXU98* was able to better adapt to the rumen environment by altering flagellar assembly and regulating the rumen microenvironment by regulating lactate and cellulase synthesis. Our research provides potential strains for improving cattle feed in practical production and provides the basis for further research into the metabolic mechanisms of different carbon sources in bacteria. In addition, further studies are needed to investigate the effect of microcrystalline cellulose on the functions of *B. licheniformis NXU98* and its underlying molecular mechanism.

## Data availability statement

The datasets presented in this study can be found in online repositories. The names of the repository/repositories and accession number(s) can be found in the article/Supplementary material.

## Ethics statement

The animal studies were approved by Ethical Review of Science and Technology of Ningxia University. The studies were conducted in accordance with the local legislation and institutional requirements. Written informed consent was obtained from the owners for the participation of their animals in this study.

## Author contributions

YC and JZ conducted the experiments, analyzed the data, and wrote the original manuscript. WR conducted parts of the experiments and analyzed parts of the data. LZ and XX conceived the research, supervised the research and revised the manuscript together. XX acquired funding. All authors contributed to the article and approved the submitted version.

## Footnotes

^1^http://rdp.cme.msu.edu/index.jsp

^2^https://cloud.majorbio.com

^3^http://www.heatmapper.ca/
