# Supplementary material for: Response of a new rumen-derived Bacillus licheniformis to different carbon sources
Source: Front Microbiol. 2023 Nov 1;14:1238767. doi: 10.3389/fmicb.2023.1238767 (PMC10646532; doi:10.3389/fmicb.2023.1238767)
Supplement: Supplementary file 1 [file Data_Sheet_1.docx]

***Supplementary Material***

**Response of a new rumen-derived *Bacillus licheniformis* to**

**different carbon sources**

**Yuchen Cheng, Jie Zhang, Wenyi Ren, Lili Zhang*** **& Xiaofeng Xu***

Yuchen Cheng and Jie Zhang contributed equally to this work.

* Correspondence:

Lili Zhang, e-mail: zhanglilinx@126.com

Xiaofeng Xu, e-mail: [xuxiaofengnd@126.com](mailto:xuxiaofengnd@126.com)

**1 Supplementary Figures and Tables**

**1.1 Supplementary Figures**


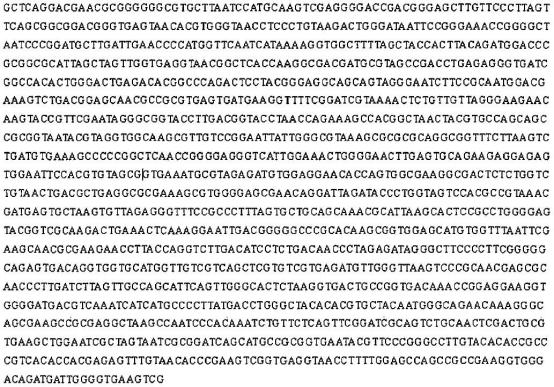


**Supplementary Figure 1. Sequencing results of 16S rDNA of selected strains**


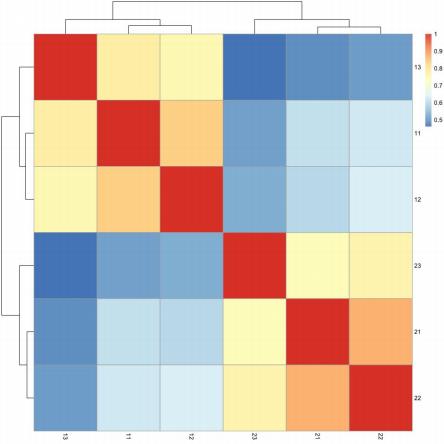


**Supplementary Figure 2. Sample correlation coefficient clustering chart.**


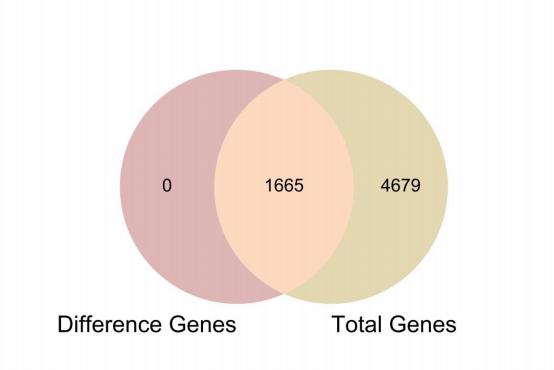


**Supplementary Figure 3. Total number of genes and significantly differentially expressed genes between different carbon sources**


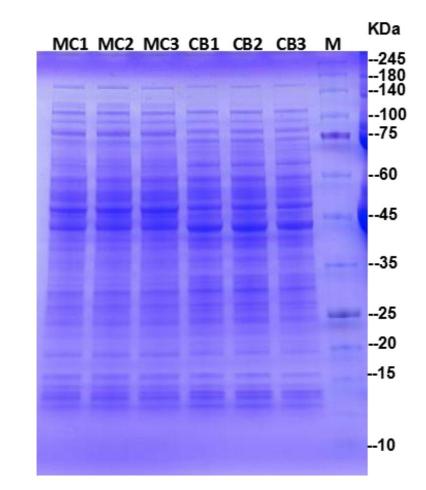


**Supplementary Figure 4. Gel graph of SDS-PAGE**


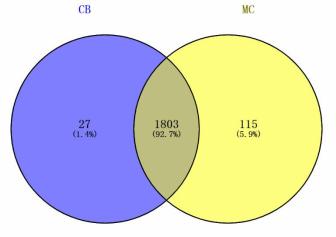


**Supplementary Figure 5. Veen map of different proteome *B. licheniformis* with different carbon source**


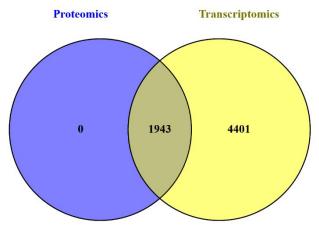


**Supplementary Figure 6. Venn map of overall gene/protein comparison of transcriptome and Proteome**

**2.2 Supplementary Table**

| Item | % of DM | | | Item | % of DM |  |
| --- | --- | --- | --- | --- | --- | --- |
|  |  |  |  |  |  |  |
| Ingredient | | | NaCl | | 0.45 |  |
| Alfalfa hay | | 10.61 | Premix^1^ | | 1 |  |
| Corn silage | | 28.92 | Nutrient composition, | | | |
| Tableting maize | | 24.75 | (MJ·kg^-1^) NE_L_^2^ | | 1.66 |  |
| Soybean meal | | 6.37 | CP | | 17.04 |  |
| Cottonseed meal | | 4.24 | EE | | 4.45 |  |
| Sprayed corn husk | | 1.06 | NDF | | 37.15 |  |
| Corn gluten meal | | 1.77 | ADF | | 21.91 |  |
| DDGS | | 3.54 | Ca | | 1.06 |  |
| Beet pellets | | 4.24 | P | | 0.49 |  |
| Cottonseed | | 6.72 |  | |  |  |
| Molasses | | 3.54 |  | |  |  |
| CaHPO3 | | 0.49 |  | |  |  |
| Limestone | | 0.52 |  | |  |  |
| NaHCO3 | | 1.36 |  | |  |  |
| MgO | | 0.42 |  | |  |  |

^1 Premix provided (per kilogram of DM)600,000 IU of vitamin A; 240,000 IU of vitamin D; 3000 IU of vitamin E;^ ^2250mg Fe;600 mg Cu;4500 mg Zn;1500 mg Mn; 150 mg l;18 mg Se; 30 mg Co.2 Calculated value (based on China NY/t 34,2004).^

**Supplementary Table 1 Composition and nutrient levels of basal diet**

| Sample_ID | Total_Reads | Total_Bases | Error% | Q20% | Q30% | GC% |
| --- | --- | --- | --- | --- | --- | --- |
| 11 | 23897732 | 3601028533 | 0.0283 | 95.93 | 92.15 | 53.04 |
| 12 | 26556530 | 4001295073 | 0.0282 | 95.95 | 92.15 | 52.61 |
| 13 | 24766696 | 3731319896 | 0.0265 | 96.86 | 93.35 | 51.89 |
| 21 | 28872316 | 4349090071 | 0.0265 | 96.83 | 93.38 | 52.54 |
| 22 | 25747756 | 3878701148 | 0.0265 | 96.89 | 93.36 | 52.03 |
| 23 | 27474706 | 4139320230 | 0.0274 | 96.36 | 92.82 | 53.72 |

**Supplementary 8. Raw data statistics. Q20, Q30%: percentage of bases with Phred values**

**greater than 20 and 30 in the total number of bases; Error%: base error rate; GC %: sum of**

**the number of bases G and C as a percentage of the total number of bases.**

|  | **MIC(E.coli)** | | | | | | | | | | | | |
| --- | --- | --- | --- | --- | --- | --- | --- | --- | --- | --- | --- | --- | --- |
|  |  | 50.0% | 25.0% | 12.5% | 6.3% | 3.1% | 1.6% | 0.8% | 0.4% | 0.2% | 0.1% | **（-）** | **（+）** |
| MC | 1 | 0.7293 | 0.7351 | 0.7249 | 0.7104 | 0.7019 | 0.6967 | 0.7491 | 0.8283 | 0.8234 | 0.8901 | 0.0773 | 0.9574 |
|  | 2 | 0.7372 | 0.6942 | 0.718 | 0.7034 | 0.7802 | 0.7329 | 0.7287 | 0.779 | 0.839 | 0.8576 | 0.0743 | 0.8847 |
|  | 3 | 0.715 | 0.7221 | 0.7406 | 0.7537 | 0.7684 | 0.7774 | 0.7826 | 0.7903 | 0.8041 | 0.8897 | 0.0777 | 0.934 |
|  |  |  |  |  |  |  |  |  |  |  |  |  |  |
|  |  |  |  |  |  |  |  |  |  |  |  |  |  |
| CB | 1 | 0.759 | 0.7714 | 0.7188 | 0.7517 | 0.7435 | 0.7844 | 0.8967 | 0.7885 | 0.9689 | 0.9906 | 0.0792 | 0.9869 |
|  | 2 | 0.7828 | 0.8063 | 0.7667 | 0.7623 | 0.794 | 0.7963 | 0.8738 | 0.8237 | 0.9623 | 0.9302 | 0.0736 | 0.9965 |
|  | 3 | 0.7581 | 0.8206 | 0.7811 | 0.791 | 0.8105 | 0.8257 | 0.8315 | 0.8101 | 0.9194 | 0.9016 | 0.0731 | 0.9185 |
|  |  |  |  |  |  |  |  |  |  |  |  |  |  |
|  |  |  |  |  |  |  |  |  |  |  |  |  |  |
|  | **MIC(S.aureus)** | | | | | | | | | | | | |
|  |  | 50.0% | 25.0% | 12.5% | 6.3% | 3.1% | 1.6% | 0.8% | 0.4% | 0.2% | 0.1% | **（-）** | **（+）** |
| MC | 1 | 0.7043 | 0.7494 | 0.7283 | 0.7354 | 0.7755 | 0.7847 | 0.8042 | 0.8136 | 0.8198 | 0.8509 | 0.0797 | 0.8868 |
|  | 2 | 0.7821 | 0.8121 | 0.8091 | 0.7883 | 0.8459 | 0.8093 | 0.8174 | 0.8505 | 0.847 | 0.9808 | 0.0628 | 0.8929 |
|  | 3 | 0.7933 | 0.8405 | 0.8212 | 0.8087 | 0.8199 | 0.8371 | 0.8483 | 0.8928 | 0.8953 | 0.8921 | 0.0744 | 0.8896 |
|  |  |  |  |  |  |  |  |  |  |  |  |  |  |
|  |  |  |  |  |  |  |  |  |  |  |  |  |  |
| CB | 1 | 0.7729 | 0.8091 | 0.8314 | 0.8278 | 0.8374 | 0.8383 | 0.8476 | 0.8475 | 0.857 | 0.8663 | 0.0672 | 0.8815 |
|  | 2 | 0.8642 | 0.8717 | 0.8775 | 0.8743 | 0.8674 | 0.8856 | 0.8717 | 0.8919 | 0.9035 | 0.9214 | 0.0692 | 0.9119 |
|  | 3 | 0.9105 | 0.9199 | 0.9707 | 0.9657 | 0.9666 | 0.9758 | 0.9674 | 0.9767 | 0.978 | 0.9847 | 0.0812 | 0.9971 |

^A.B.C^ Means within the same row with no common superscript differ significantly (*P*< 0.05).

**Supplementary** **9 MIC of NXU 98 against different bacteria**
